# Strategies for launching streaming content: Assessing movie-country relatedness and its impact on international popularity

**DOI:** 10.1371/journal.pone.0305433

**Published:** 2024-06-14

**Authors:** Changjun Lee, Sung Wook Ji

**Affiliations:** 1 School of Convergence, Sungkyunkwan University, Seoul, South Korea; 2 Department of Media Communication, Hankuk University of Foreign Studies, Seoul, South Korea; Rikkyo University, JAPAN

## Abstract

The study proposes a way to measure the relatedness between countries and movies (movie—country relatedness density) and to test whether this relatedness is an important factor in predicting the popularity of a movie in a certain country. The results show that both movie—country relatedness density and movie ubiquity (i.e., popularity across many countries) are positively associated with a movie appearing in a country’s top 20 list even after considering other covariates. Based on these findings, we suggest an OTT (Over-the-tops) movie launching strategy with regard to movie—country relatedness density for both global and local OTT companies. Our study contributes to a growing body of research on movie consumption patterns and provides insights into the factors that determine a movie’s success on a global scale. The importance of cultural similarity in adopting films and television shows is widely recognized, but the concept of movie—country compatibility is introduced to take into account the content attributes that cannot be explained by cultural differences. The results suggest that movies that are related to other popular movies in a given country and movies that are popular across many countries are more likely to appear in a country’s top 20 list.

## Introduction

The emergence of global OTTs (Over-the-tops) has revolutionized the media industry, allowing audiences to access a wide range of content from different countries and cultures. These services, such as Netflix and Disney+ has lowered the cultural barriers associated with media consumption and created new opportunities for cross-cultural understanding. In 2022, Korean television (TV) shows like *Squid Game* and *Extraordinary Attorney Woo* overcame cultural differences to find success worldwide [1]. Similarly, *Strange Things* attracted a massive global fanbase despite its being set in the United States during the 1970s and 1980s [2].

While these examples suggest that cultural barriers are being lowered, achieving global success is still a challenge for many TV shows and global mega-hit often comes down to serendipity. Despite the availability of global OTT platforms, there are still cultural barriers that can make it difficult for some shows to gain popularity among audiences in different countries. Additionally, cultural preferences and tastes can vary widely across different regions and countries, making it challenging for producers to create content that appeals to a global audience.

In countries where global OTTs have been introduced, local media channels have become threatened. One of the biggest challenges is competition from global OTT players such as Netflix, Amazon Prime Video, and Disney+. These global players have established a strong presence in the market and offer a wide range of content that appeals to audiences worldwide. As a result, local OTT providers have emerged to cater to the specific needs of local audiences and compete with global players. Such local providers face two main challenges: producing culturally appropriate content that has the potential to become a global mega-hit and renting only the overseas content that appeals to local users [3]. While the latter concern is about purchasing priority and cost-effectiveness, the former involves creating high-quality content that resonates with users worldwide [4]. Local OTT operators who are latecomers to the market require additional strategies to launch high-quality content that caters to the preferences of local users.

Previous research has explored various aspects of OTT launching strategies. Park and Kwon [5] analyzed the strategies used by major broadcasting countries and OTT operators and found that both groups employ similar strategies, such as localization, partnership, content differentiation, revenue enhancement, and service optimization. Local OTT providers have adopted a multi-faceted approach for launching their services in major countries that incorporates these strategies. Wang and Jung [3] noted that local OTT providers may assess the demand for their platform and establish partnerships with foreign OTT players, with a focus on acquiring content that is of the same genre and quality as that offered by Netflix and Disney Plus. This enables local OTT providers to offer a comparable level of content and quality to their subscribers and position themselves as a viable alternative to these established players. Sharma and Lulandala [6] suggested that local OTT providers can increase their chances of success by developing local content repositories using local/regional languages to serve rural niche markets, and by forming strategic collaborations with mobile network companies, financial institutions, and technology companies to boost subscriptions and improve the consumer experience. However, there is still limited understanding of OTT launching strategies that take into account the compatibility between movies and countries.

Jang et al. [7] have conducted research that highlights the significance of linguistic proximity, geographical distance, and cultural difference in determining content consumption similarity between countries. This research raises important questions about the relationship between content and countries and its impact on the popularity of new entries in a given country. Accurate measurements of the relatedness between content and countries are crucial in understanding the factors that influence content consumption patterns. If such measurements were available, it would be possible to develop a more comprehensive and tailored approach to creating culturally relevant content that could be used by local OTT providers. This would allow them to better understand the needs and preferences of local audiences, and offer content that is relevant, appealing, and engaging. The ability to tailor content to local audiences would likely increase the chances of success for local OTT providers, as it would help them to stand out from the competition and establish themselves as a viable alternative to global players.

This study proposes a way to measure the relatedness between countries and movies (i.e., movie—country relatedness density) and to test whether this relatedness is an important factor in predicting the popularity of a certain movie in a certain country. Based on the findings, we suggest a local OTT strategy that is tailored to movie—country relatedness. Our study contributes to a growing body of research on movie consumption patterns and provides insights into the factors that determine a movie’s success on a global scale.

## Theoretical review

It is difficult to analyze the cultural industry because cultural goods cannot be easily expressed as a utility function [8]. The cultural industry encompasses a wide range of sectors, including media (e.g., films, television, music recording, publishing), fashionable consumer goods (e.g., clothing, furniture, jewelry), services (e.g., advertising, tourism, entertainment), creative professions (e.g., architecture, graphic arts, webpage design), and collective cultural consumption institutions (e.g., museums, art galleries, concert halls [9]).

Importing (and exporting) films or other types of content is a sensitive issue in international trade because content carries cultural messages [10]. Many countries have various systems to protect their local content from that of other markets, especially Hollywood, because policymakers believe that cultural economy is more than a set of diverse industries. Cultural goods are deeply rooted in the place where they are produced [11]. Commodified cultural goods, such as films, TV shows, music, and fashion, reflect the local image, cultural context, and lifestyle. Cultural economy and place coevolve and are influenced by globalization and multicultural companies such as Disney and Sony. Therefore, multinational (and culturally diverse) content on OTT has both global and local properties. This highlights OTT companies’ need for a new content launching strategy that considers the characteristics of cultural goods [6].

Scholars agree on the importance of cultural similarity in countries’ decisions to adopt films and TV shows from other places [4,6,7,12]. However, due to content globalization, many cultural attributes are shared between countries, and there is a special content—country compatibility due to content attributes that cannot be explained by cultural differences. To test this hypothesis, we borrow the concept of product—country compatibility from the field of economic geography [13] to create a new index for content—country compatibility.

Product-country compatibility is an established concept in the field of economic geography that explores the extent to which a product is well-suited to the cultural, economic, and regulatory conditions of a specific country [14]. The alignment of a product with the cultural norms, values, economic conditions, and regulatory framework of a target country can significantly impact its success in that market. According to Jun et al. [15], the compatibility between a product and a target country can be evaluated through the concept of relatedness, which encompasses three dimensions: product relatedness, importer relatedness, and exporter relatedness. Product relatedness refers to the extent to which the target country already exports similar products to the destination country. Importer relatedness considers whether the target country exports the same product to neighboring countries of the destination country. Exporter relatedness examines whether neighboring countries of the target country are already exporting the same product to the destination country. The assessment of these three dimensions of relatedness provides a comprehensive evaluation of the compatibility between a product and a target country, and can help to determine the potential for success in that market.

The significance of product-country compatibility has been widely recognized in the field of international business and economic geography [16]. Empirical evidence suggests that products that are culturally aligned with a target market are more likely to achieve success, as consumers in that market are more likely to connect with the product and perceive it as relevant to their needs and preferences [17]. Furthermore, products that are compatible with a target country’s economic and regulatory environment are more likely to be profitable, as they can be priced competitively in that market and are less likely to face regulatory hurdles. These findings highlight the crucial role that product-country compatibility plays in determining the success of a product in a foreign market.

There are several factors that can affect product—country compatibility. For example, cultural factors such as language, religion, and social norms can influence whether a product is perceived as appropriate for a given market [18]. Economic factors such as consumer income and market demand can also impact the compatibility of a product, as can regulatory factors such as product safety standards and import/export regulations.

The concept of product-country compatibility is well demonstrated through the example of Halal-certified food products. Halal certification ensures that a product is compliant with Islamic dietary laws, which can be a critical consideration for Muslim consumers. Hence, companies that produce Halal-certified food products are more likely to experience success in Muslim-majority countries where there is a strong demand for Halal food. Conversely, a product that is not compatible with a target country’s cultural or regulatory environment may encounter difficulties in gaining acceptance in that market, highlighting the crucial role that product-country compatibility plays in determining the success of a product in a foreign market.

Content—country compatibility refers to the degree to which a movie or TV show is suitable for the cultural, economic, and regulatory conditions of a particular country. The compatibility of a movie or TV show with the country in which it is being distributed can have a significant impact on its success in that market. For example, movies or TV shows that are well suited to a country’s cultural norms and values are more likely to be popular among local audiences. Similarly, if a movie or TV show is compatible with a country’s economic and regulatory conditions, it is more likely to be profitable for the company distributing it.

The importance of content—country compatibility is similar to that of product—country compatibility. Research has shown that content that aligns with a country’s cultural norms and values is more likely to be successful among local audiences [6,7]. For example, movies or TV shows that feature local actors, settings, or themes may be more appealing to audiences in a given country. Furthermore, content that aligns with a country’s economic and legal framework is more likely to be profitable, as it can be offered at a price that is competitive in that market and has a lower risk of facing regulatory obstacles.

Product-country compatibility is influenced by a complex interplay of cultural, economic, and regulatory factors. Cultural factors, such as language, humor, and storytelling style, play a critical role in shaping the perception of a movie or TV show in a given market. Economic factors, such as the level of disposable income and the accessibility of streaming services, can also impact the compatibility of a movie or TV show with a target market. Furthermore, regulatory factors, such as censorship laws and import/export regulations, can significantly influence the distribution and success of content in a target country. These factors highlight the intricate nature of product-country compatibility and the need to consider a range of cultural, economic, and regulatory factors when evaluating the potential success of a product in a foreign market.

In sum, content—country compatibility is an important concept for movie and TV show producers and distributors to consider when expanding to new markets. Understanding the degree of compatibility between content and the country in which it will be distributed is essential for creating successful and profitable products. By aligning content with a country’s cultural, economic, and regulatory conditions, companies can increase the likelihood of success in their international business endeavors.

To measure content—movie compatibility in this study, we use the relatedness density index. The related density for a specific piece of content (i.e., film or TV show) in a specific region is the region’s relative advantage of the relatedness between the content and all other content in a local level divided by the relatedness among all content in a global scale [19,20]. This calculation incorporates ∅_*i*,*j*_, a crucial component representing the relatedness between content *i* and *j* by considering their co-occurrence within top-tiered lists, thereby capturing the extent to which two pieces of content share contextual or thematic similarities that resonate across different geographical regions. Eq (1) is the mathematical calculation of relatedness between content *i* and *j*:

$$\emptyset_{i,j}=\frac{N_{i,j}}{N_{i}∙N_{j}}$$ (1)

where *N*_*i*,*j*_ is the number of times content *i* and *j* co-occur in top-tiered lists, *N*_*i*_ and *N*_*j*_ are the total occurrences of content I and j, respectively, across all top-tiered lists.

Eq (2) represents how to measure content—country related density. *RD*_*r*,*i*_ denotes the related density of region *r* to content *i*. ∅_*i*,*j*_ denotes the relatedness between content *i* and *j* measured by considering the co-occurrence of top-tiered lists.


$${RD}_{r,i}=\frac{\sum_{j\in r,\;j\neq i}\emptyset_{i,j}}{\sum_{j\neq i}\emptyset_{i,j}}\times 100$$ (2)

Movies and TV shows are known to exhibit variation in elements such as themes, characters, and cultural references. These elements can greatly impact the level of compatibility between a movie or TV show and a specific country, as countries have diverse cultural norms, values, and preferences [8]. This study’s first hypothesis posits that a higher degree of compatibility between a movie or TV show and a country results in a greater level of interest and engagement by audiences in that country. This increased interest and engagement in turn increases the probability of the movie or TV show’s inclusion in the top 20 list of popular content in that country. Hence, the compatibility between movies and TV shows and different countries predicts the likelihood of these movies and TV shows’ inclusion in the top 20 list of popular content in a specific country.

***H1***: Movies and TV shows exhibit varying degrees of compatibility with different countries, and this compatibility predicts a given country’s likelihood of including that content in its top 20 list.

The compatibility between content and country is widely recognized as a crucial factor in determining the popularity of movies and TV shows in a particular country. However, global mega-hit content that becomes popular across a wide range of countries, regardless of its compatibility with any specific country, may occasionally emerge. This phenomenon of widespread popularity can be attributed to various factors such as effective marketing campaigns, favorable media coverage, and word-of-mouth recommendations. This study’s second hypothesis posits that the ubiquity of such popular content, despite the content’s incompatibility with a given country, also predicts the likelihood of that content’s inclusion in the top 20 list of popular content in that country.

***H2***: Despite the importance of content—country compatibility, global mega-hit content that achieves widespread popularity regardless of its compatibility with a particular country may occasionally emerge. This ubiquity of popular content predicts a given country’s likelihood of including that content in its top 20 list.

## Method

In conducting our analysis, we used data on the monthly top 20 movies in 80 countries from January 2021 to December 2021, which we obtained from the Flixpatrol website (https://flixpatrol.com). This website provides daily, weekly and monthly rankings and statistics on movies and TV shows available on popular OTT platforms like Netflix, Amazon Prime Video, Disney+, and etc.. The website tracks the popularity of titles on a daily basis and updates its rankings accordingly, which allowed us to collect the data of monthly ranking of movie titles in specific countries. Among all OTTs, we use Netflix data as a global OTT service provider because it is a valuable source for understanding movie consumption patterns on a global scale. The final sample included 1,939 movies that appeared at least once in any country’s top 20, resulting in a total of 1,861,440 observations.

Eq (3) is an econometric model we used to test our hypotheses. In particular, it is a linear regression model that aims to examine the impact of country—movie relatedness density (*RD*_*c*,*m*,*t*_), movie ubiquity (*U*_*m*,*t*_), other covariates (*Z*_*m*,*t*_), and monthly fixed effects (*Mon*_*t*_) on the entry of a movie into the top 20 list of a given country at period *t+1*.


$${ENT\_TOP20}_{c,m,t+1}=\beta_{1}{RD}_{c,m,t}+\beta_{2}U_{m,t}+\beta_{3}Z_{m,t}+\beta_{4}{Mon}_{t}+\epsilon_{c,m,t}$$ (3)

The dependent variable in our analysis was the new entry of a movie *m* in the top 20 of a country *c* in the period *t+1*, denoted as *ENT_TOP*20_*c*,*m*,*t*+1_. Our independent variables included country—movie relatedness density and movie ubiquity. Relatedness density, denoted by *RD*_*c*,*m*,*t*_, is a measure of the relatedness between country *c* and movie *m* at period *t*. High relatedness density indicates that movie *m* is not currently on the list of top 20 films in country *c*, but there are many movies similar (or related) to movie *m* on the current list of the top 20 in country *c* at period *t*. Movie ubiquity, denoted by *U*_*m*,*t*_, is a measure of the popularity of movie *m* at period *t* all over the world. We measured this variable by counting the number of top 20 movie lists across countries on which movie *m* was listed at period *t*. With a potential maximum value of 80, we applied min-max normalization to facilitate direct comparison of coefficient magnitudes with those of the relatedness density.

Furthermore, our model incorporates a range of covariates to control for additional influences on a movie’s likelihood of ranking within a country’s top 20. These include characteristics specific to each movie and period (***Z***_*m*,*t*_), such as its status as a Netflix Original, the country of production, genre, Rotten Tomatoes score, and IMDB (Internet Movie Database) score. These factors are critical in adjusting for the impact of a movie’s genre and its reception among critics and audiences, which can significantly affect its international popularity.

**Fig 1:**
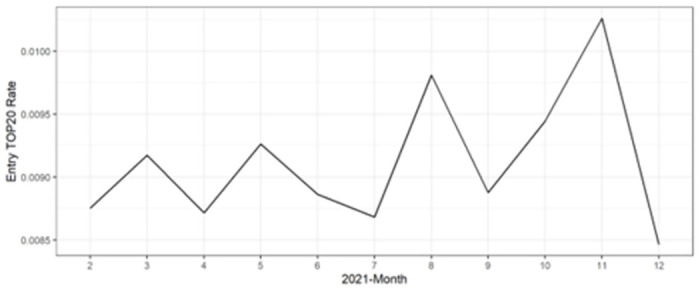
Top 20 entry rate of 1,372 movies in the sample by month. **Note**: Nine out of 1,000 (0.9%) movies, on average, were newly listed in the top 20 in 2021.

Fig 1 presents the changes in the monthly average values of the dependent variable, Entry of Top 20, for the study sample (comprising 1,372 movies). On average, 0.9% (nine out of 1,000) movies were newly listed in the top 20 in 2021. A high value signifies that a considerable number of films from the sample entered the top 20, while a low value indicates that fewer films made it into the top 20. In essence, a low Entry of Top 20 rate implies that a limited number of content pieces gained popularity across multiple countries. For instance, December exhibits the lowest rate, which can be attributed to the simultaneous global popularity of Christmas-themed content within the top 20.

**Fig 2:**
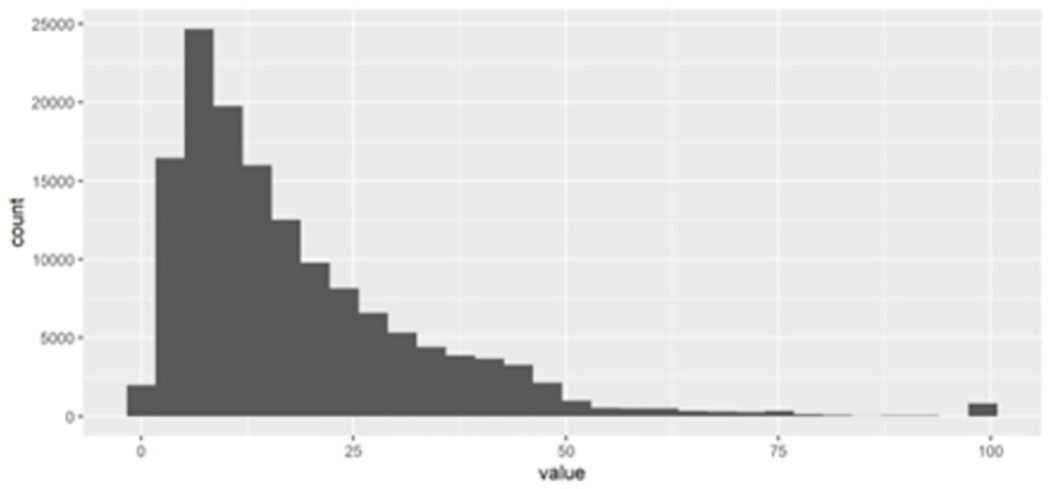
Histogram of country–movie relatedness density.

**Fig 3:**
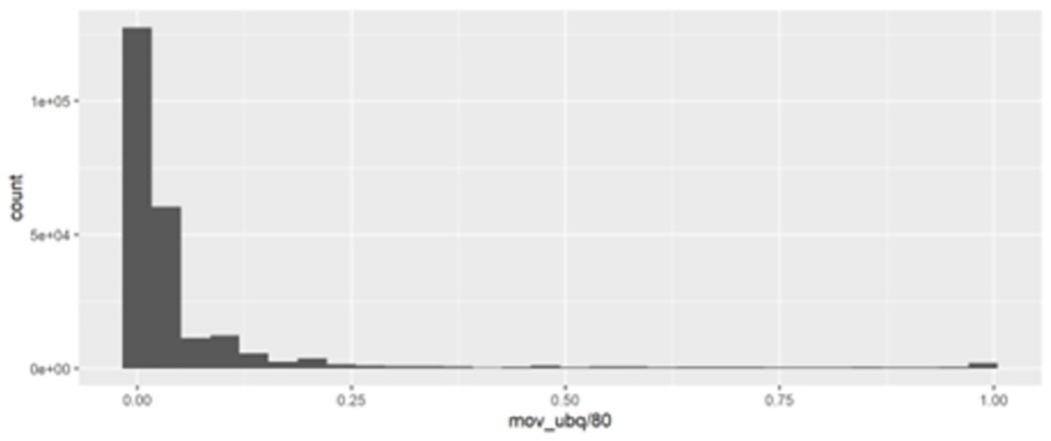
Histogram of movie ubiquity.

Fig 2 displays the histogram of country-movie relatedness density, which is the most crucial independent variable. The overall distribution is right-skewed, and a high value suggests a strong, enduring relationship between a country and a specific movie. Fig 3. illustrates the distribution of the independent variable, movie ubiquity, which is more right-skewed than the country-movie relatedness density.

Table 1 presents descriptive statistics for the variables used in the study. The *TOP20 Dummy* variable is a binary variable that takes the value of 1 if a movie appeared in a country’s top 20 at period *t* and 0 otherwise. The variable is highly skewed, with only 1.0% of the observations having a value of 1. The *Entry Dummy* variable is also binary and takes the value of 1 if a movie entered a country’s top 20 for the first time in the next period and 0 otherwise. Similar to the *TOP20 Dummy* variable, it is highly skewed, with only 0.9% of the observations having a value of 1. The *Netflix Original* variable is a binary variable that takes the value of 1 if a movie is a Netflix Original and 0 otherwise. The variable indicates that 12.8% of the movies in the study are Netflix Originals.

**Table 1:** Descriptive statistics of variables in use in the final sample.

Var.	Levels	Stats
cid (country ID)	80 unique values	
mid (Movie ID)	1939 unique values	
TOP20 dummy	0	1688923 (99.0%)
	1	17397 (1.0%)
Entry dummy	0	1690759 (99.1%)
	1	15561 (0.9%)
Netflix Original	0	1487200 (87.2%)
	1	219120 (12.8%)
Related density	Mean ± SD	0.2 ± 0.2
Movie Ubiquity	Mean ± SD	0.0 ± 0.1
IMDB Score	Mean ± SD	6.3 ± 1.1
Rotten Tomato Score	Mean ± SD	53.6 ± 25.9
Genre	Action	196240 (11.5%)
	Adventure	44880 (2.6%)
	Animated	118800 (7.0%)
	Comedy	293920 (17.2%)
	Crime	66880 (3.9%)
	Documentary	39600 (2.3%)
	Drama	190080 (11.1%)
	Etc	438240 (25.7%)
	Romance	81840 (4.8%)
	Science Fiction	86240 (5.1%)
	Thriller	149600 (8.8%)
Producing country	Etc	478720 (28.1%)
	France	51040 (3.0%)
	Germany	24640 (1.4%)
	India	45760 (2.7%)
	Japan	43120 (2.5%)
	Nigeria	29920 (1.8%)
	South Korea	41360 (2.4%)
	Spain	27280 (1.6%)
	United Kingdom	75680 (4.4%)
	United States	888800 (52.1%)
Month in 2021	FEB	155120 (9.1%)
	MAR	155120 (9.1%)
	APR	155120 (9.1%)
	MAY	155120 (9.1%)
	JUN	155120 (9.1%)
	JUL	155120 (9.1%)
	AUG	155120 (9.1%)
	SEP	155120 (9.1%)
	AUG	155120 (9.1%)
	NOV	155120 (9.1%)
	DEC	155120 (9.1%)

The *Related Density* variable represents the country—movie relatedness density. It has a mean of 0.2 and a standard deviation of 0.2. The variable ranges from 0 to 1, with higher values indicating greater relatedness between a movie and a country’s current top 20. The *Movie Ubiquity* variable represents the popularity of a movie across the world. It has a mean of 0.0 and a standard deviation of 0.1. The variable ranges from 0 to 80, with higher values indicating that a movie appears in more top 20 lists.

The *IMDB Score* represents the IMDB score of a movie. It has a mean of 6.3 and a standard deviation of 1.1. The *Rotten Tomatoes Score* is the Rotten Tomatoes score of a movie. It has a mean of 53.6 and a standard deviation of 25.9. *Genre* represents the genre of a movie, with several categories such as *Action*, *Comedy*, *Drama*, and *Science Fiction*. The *Etc* category indicates other genres that are not listed. The *Producing Country* variable represents the country where a movie was produced. Finally, the *Month in 2021* variable indicates the month in 2021 when a movie appeared in a country’s top 20. Each month has an equal number of observations (9.1% of the total sample).

Table 2 shows the descriptive statistics of the key independent variables (*Related Density*, *Movie Ubiquity*, *IMDB Score*, *Rotten Tomatoes Score*, *Netflix Original*, *Genre*, and *Producing Country*) by the entry status of the given movie (Entry = 0, Entry = 1). Among the 1,706,320 observations, 15,561 (0.9%) movies newly entered the top 20 list in a given country at period *t+1*. The mean value of *Related Density* was higher for Entry = 1 (0.5 ± 0.2) compared to Entry = 0 (0.2 ± 0.2), and the difference was statistically significant (*p* < .001). The mean value of *Movie Ubiquity* was also higher for Entry = 1 (0.4 ± 0.4) compared to Entry = 0 (0.0 ± 0.0), and the difference was statistically significant (*p* < .001).

**Table 2:** Comparative statistics of variables in use by the dependent variable (entry).

Var.	Levels	Entry = 0 (N = 1,690,759)	Entry = 1 (N = 15,561)	Sig. Diff (p-value)
Related density	Mean ± SD	0.2 ± 0.2	0.5 ± 0.2	<.001
Movie Ubiquity	Mean ± SD	0.0 ± 0.0	0.4 ± 0.4	<.001
IMDB	Mean ± SD	6.4 ± 1.1	6.3 ± 1.0	<.001
Rotten	Mean ± SD	53.6 ± 25.9	53.6 ± 26.2	.856
Netflix Original	0	1478616 (87.5%)	8584 (55.2%)	<.001
	1	212143 (12.5%)	6977 (44.8%)	
Genre	Action	194049 (11.5%)	2191 (14.1%)	<.001
	Adventure	44209 (2.6%)	671 (4.3%)	
	Animated	117181 (6.9%)	1619 (10.4%)	
	Comedy	291354 (17.2%)	2566 (16.5%)	
	Crime	66324 (3.9%)	556 (3.6%)	
	Documentary	39272 (2.3%)	328 (2.1%)	
	Drama	188705 (11.2%)	1375 (8.8%)	
	Etc	435456 (25.8%)	2784 (17.9%)	
	Romance	80986 (4.8%)	854 (5.5%)	
	Science Fiction	85354 (5%)	886 (5.7%)	
	Thriller	147869 (8.7%)	1731 (11.1%)	
Producing country	Etc	476365 (28.2%)	2355 (15.1%)	<.001
	France	50398 (3%)	642 (4.1%)	
	Germany	24273 (1.4%)	367 (2.4%)	
	India	45511 (2.7%)	249 (1.6%)	
	Japan	42856 (2.5%)	264 (1.7%)	
	Nigeria	29871 (1.8%)	49 (0.3%)	
	South Korea	41131 (2.4%)	229 (1.5%)	
	Spain	26914 (1.6%)	366 (2.4%)	
	United Kingdom	75066 (4.4%)	614 (3.9%)	
	United States	878374 (52%)	10426 (67%)	

The mean value of *IMDB Score* was not statistically different between the Entry = 0 (6.4 ± 1.1) and Entry = 1 (6.3 ± 1.0) groups. The mean value of *Rotten Tomatoes Score* was also not significantly different between the two groups (*p* = .856). The proportion of Netflix Original movies was higher for Entry = 1 (44.8%) compared to Entry = 0 (12.8%), and the difference was statistically significant (*p* < .001). The proportion of movies in each genre and producing country was also different between the two groups, and the differences were statistically significant (*p* < .001). In sum, we found that there were significant differences in *Related Density*, *Movie Ubiquity*, and *Rotten Tomatoes* score by the dependent variable, entry.

## Results

Table 3 shows the results of three different models analyzing the relationship between the dependent variable *ENT_TOP*20_*c*,*m*,*t*+1_ and the independent variables. Model 1 has only two independent variables, *Related Density* and *Movie Ubiquity*, while Model 2 and Model 3 include those two variables plus other independent variables such as *IMDB Score*, *Rotten Tomatoes Score*, *Netflix Original*, *Genre*, *Producing Country*, and *Monthly Fixed Effects*.

**Table 3:** Regression results of movie entry into Top 20 based on relatedness density, movie ubiquity, and movie characteristics.

Var.	*Model 1*			*Model 2*			*Model 3*		
	Coef.	(S.E.)		Coef.	(S.E.)		Coef.	(S.E.)	
Related density				6.12	(0.04)	***	6.2	(0.04)	***
Movie Ubiquity				9.11	(0.05)	***	10.12	(0.06)	***
**Movie specifics**									
IMDB score	0.005	(0.01)					0.08	(0.01)	***
Rotten T score	-0.001	(0.00)	**				0.001	(0.00)	***
Netflix original	1.95	(0.02)	***				-1.01	(0.04)	***
**Genre**									
Adventure	-0.02	(0.05)					0.19	(0.07)	**
Animated	0.03	(0.03)					-0.15	(0.05)	**
Comedy	-0.45	(0.03)	***				-0.11	(0.04)	*
Crime	-0.36	(0.05)	***				0.17	(0.06)	**
Documentary	-1.32	(0.06)	***				0.57	(0.07)	***
Drama	-0.56	(0.04)	***				-0.01	(0.05)	
Etc	-0.31	(0.03)	***				0.1	(0.04)	*
Romance	-0.18	(0.04)	***				0.04	(0.06)	
Science Fiction	-0.29	(0.04)	***				0.19	(0.05)	***
Thriller	-0.15	(0.03)	***				-0.11	(0.05)	*
**Producing Country**									
France	0.67	(0.05)	***				0.42	(0.07)	***
Germany	0.97	(0.06)	***				0.26	(0.09)	**
India	-0.02	(0.07)					0.75	(0.07)	***
Japan	-0.14	(0.07)	*				0.57	(0.08)	***
Nigeria	-0.84	(0.14)	***				-0.45	(0.15)	**
South Korea	0.04	(0.07)					0.11	(0.10)	
Spain	0.31	(0.06)	***				0.62	(0.09)	***
UK	0.6	(0.05)	***				0.07	(0.06)	
United States	0.98	(0.02)	***				0.47	(0.03)	***
(Intercept)	-5.55	(0.06)	***	-7.48	(0.02)	***	-8.23	(0.10)	***
Monthly F.E.	Yes			Yes			Yes		
Observations	1,706,320			1,706,320			1,706,320		
AIC	76330			79861			78206		

**Notes**: The asterisks indicate the level of statistical significance of the coefficient estimate, with more asterisks indicating greater significance (*** indicates p<0.001, ** indicates p<0.01, and * indicates p<0.05).

In all three models, both *Related Density* and *Movie Ubiquity* are positively associated with the dependent variable. This suggests that movies that are related to other popular movies in a given country, as well as movies that are popular in many countries, are more likely to appear in that country’s top 20 list.

In Model 2 and Model 3, several other independent variables are also significant predictors of the dependent variable. For example, a higher IMDB score is positively associated with appearing in the top 20, while a higher Rotten Tomatoes score is negatively associated. Netflix Original status is positively associated with appearing in the top 20 in Model 1 but negatively associated in Model 3. The *Genre* and *Producing Country* variables also show significant variation in their coefficients across the three models.

Considering the Akaike Information Criterion (AIC) values, Model 1 exhibits the lowest AIC, indicating it is the best fit, whereas Model 2 presents the highest AIC, signifying it is the least appropriate fit. Model 3, which integrates both relatedness density and movie ubiquity with other significant predictors, exhibits a balance between model complexity and predictive power. This suggests that while adding these variables increases the model’s complexity, it also enhances its ability to accurately predict a movie’s success across different countries.

Overall, these results suggest that while a movie’s relatedness to other popular movies in a country and its ubiquity across countries are important factors in predicting the movie’s likelihood of appearing in a given country’s top 20 list, there are also other important factors to consider.

## Discussion

The main findings of this study are as follows. The results show that both *Related Density* and *Movie Ubiquity* are positively associated with the dependent variable in all three models even after considering all the covariates that may cause bias. This implies that movies that are related to other popular movies in a given country and movies that are popular across many countries are more likely to appear in a country’s top 20 list. Therefore, our hypotheses are supported. The degree to which a movie or TV show is suitable for different countries can affect the likelihood of it appearing in that country’s top 20 list. However, even if a particular piece of content is not well suited for a specific country, it may still become a global phenomenon and be highly likely to appear in the top 20 list of that country due to its widespread popularity.

The second and third models indicate that other variables such as *IMDB Score*, *Rotten Tomatoes Score*, *Netflix Original*, *Genre*, *Producing Country*, and *Monthly F*.*E*. are significant predictors of the dependent variable. For instance, a higher IMDB score is positively associated with appearing in the top 20, while a higher Rotten Tomatoes score is negatively associated. Being a Netflix original is positively associated with appearing in the top 20 in Model 1 but negatively associated in Model 3. The *Genre* and *Producing Country* variables also show significant variation in their coefficients across the three models.

In summary, the study shows that a movie’s relatedness to other popular movies in a country and its ubiquity across countries are significant predictors of its likelihood of appearing in a given country’s top 20 list. However, there are other important factors that also affect a movie’s appearance on the top 20 list.

**Fig 4:**
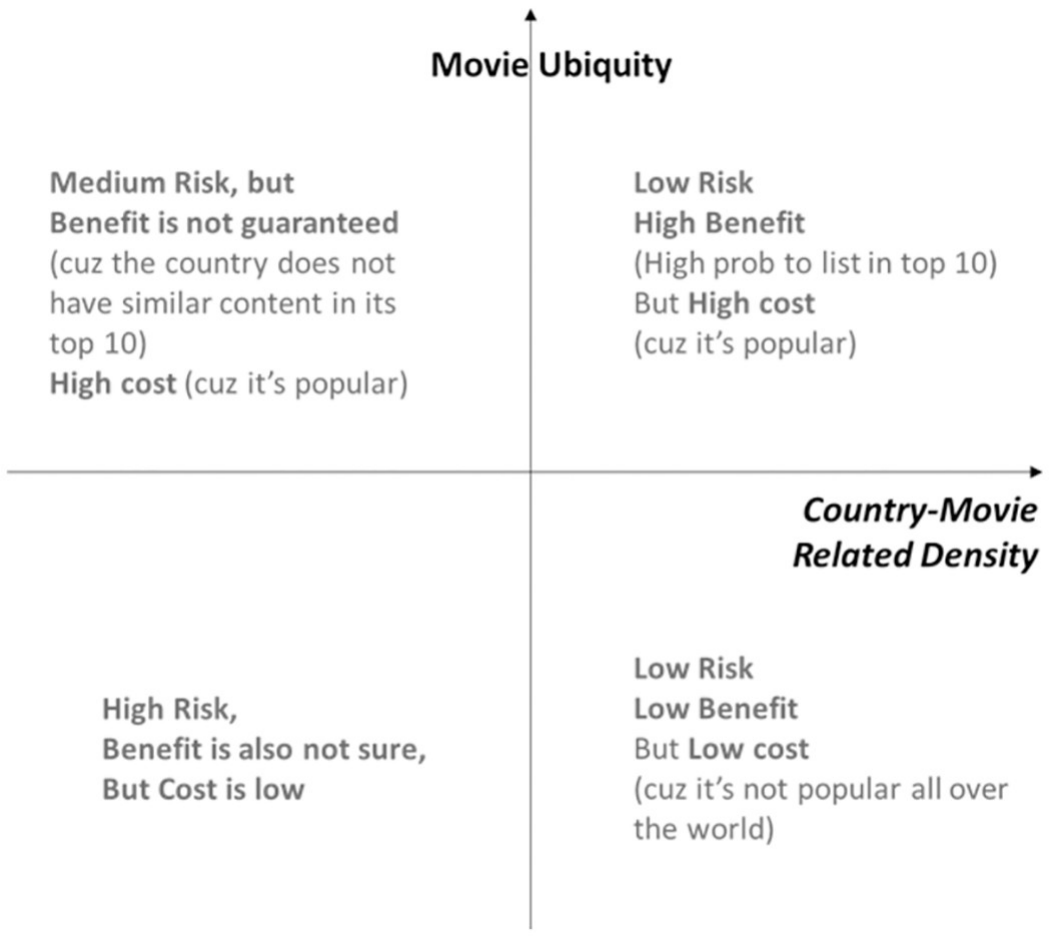
OTT content launching strategy map.

Based on the findings, we recommend for both global and local OTT companies an OTT movie launching strategy that takes into account country—movie related density. Fig 4. presents the OTT content launching strategy map, with the *x*-axis representing country—movie related density and the *y*-axis representing movie ubiquity. All movie content can be categorized within this framework. Movies that have both high related density and high movie ubiquity belong to the low-risk, high-benefit section (Quadrant 1). If local OTT providers choose to launch these types of movies, the movies may require a significant investment, but the likelihood of achieving a return on the investment is high due to the movies’ popularity across the world.

In contrast, there are instances where movie ubiquity is high but related density is low (Quadrant 2). Despite their popularity, these movies may have limited appeal to local users, making them a moderate-risk option. Meanwhile, there are also cases where movie ubiquity is low but related density is high (Quadrant 4). These movies may have limited popularity, but their costs to launch are low and expected profits are also low. However, due to their high compatibility with local user preferences, they may nevertheless yield unexpected profits.

**Fig 5:**
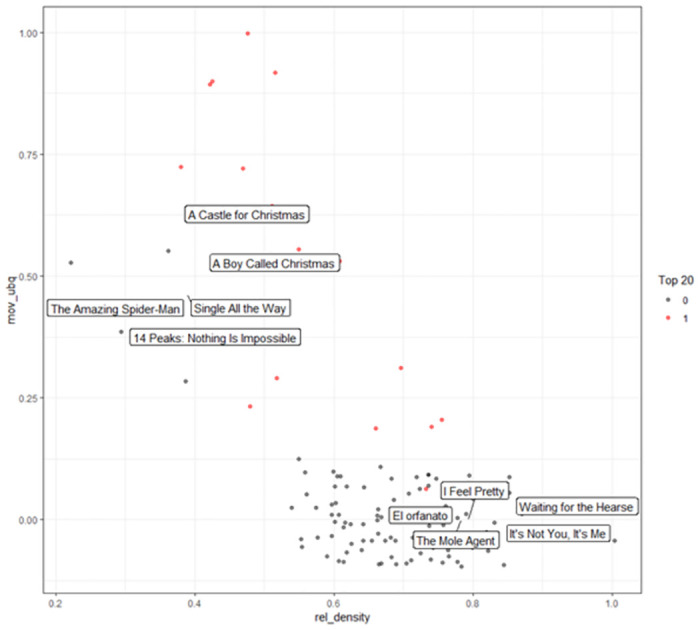
An example of an OTT content launching strategy map for Argentina, December 2021.

Fig 5 presents a visualization of Argentina’s OTT content launching map for December 2021 using the proposed framework. The red dots represent content that is already in the top 20 list, while the black dots indicate content that has not yet entered the top 20 list. Content in the first quadrant is recommended first, followed by content with high related density or movie ubiquity in the second and third quadrants. The selection criterion is the sum of the related density and ubiquity values as the score, and the scores are recommended in order of high score, and a label is attached to the figure. In other words, a combination of related density and ubiquity values are added together to create a score, and figures with higher scores are recommended. Examples of famous movies with low local compatibility include *A Castle for Christmas*, *A Boy Called Christmas*, and *Single All the Way*. Given the time of year of this map, Christmas-related content is prevalent. On the other hand, locally compatible but less well-known content such as *I Feel Pretty* and *Waiting for the Hearse* is also recommended.

The strategic takeaway from this visual analysis is to not only prioritize content that is already popular but also to identify and cultivate films with strong local relevance that have yet to break into the top rankings. By considering the sum of related density and ubiquity scores, OTT platforms can develop a more nuanced content strategy that leverages both globally successful titles and those with untapped potential within specific markets, like Argentina.

**Fig 6:**
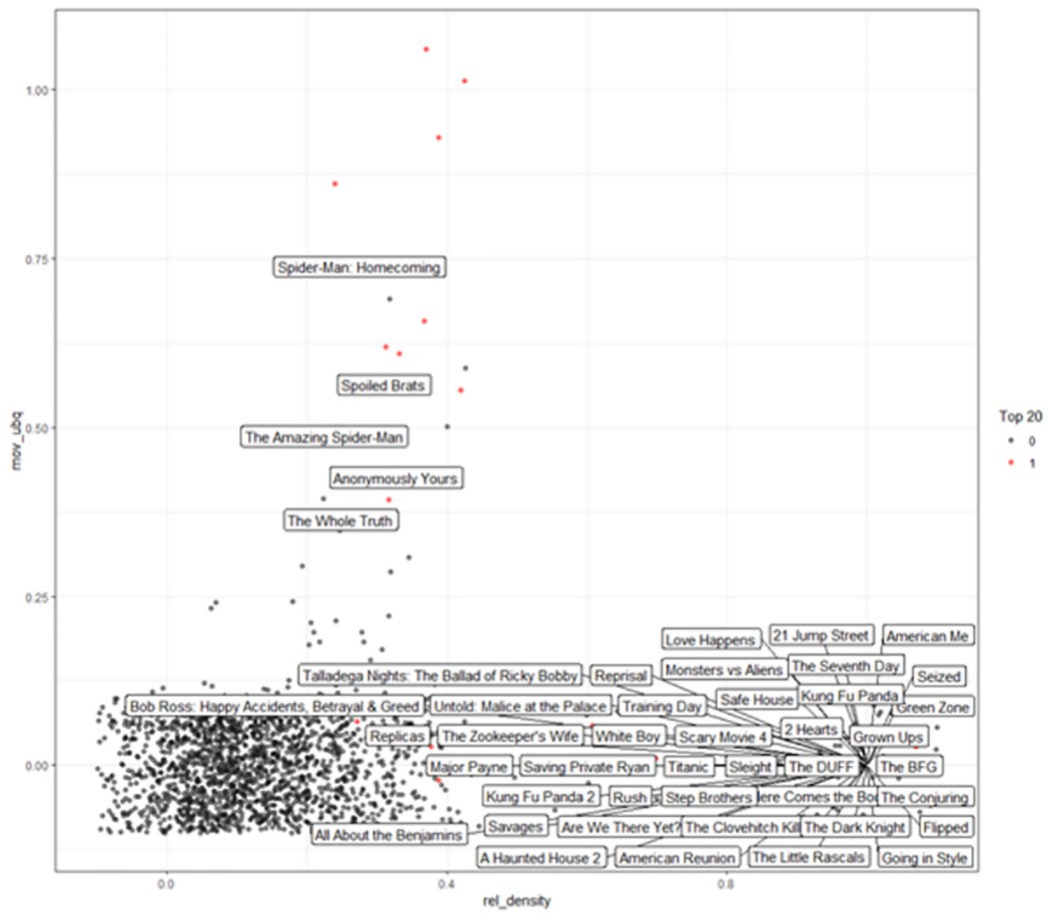
Recommendation to TOP20 High probability of entry in the United States, December 2021.

Fig 6 presents a visualization of the United States’ OTT content launching map for December 2021 using the proposed framework. The red dots, denoting films that have made it into the top 20, are primarily concentrated towards the higher end of both axes, suggesting a strong correlation between a movie’s relatedness density and ubiquity and its popularity. Notably, films such as "Spider-Man: Homecoming" and "The Amazing Spider-Man" show high ubiquity but lower relatedness density, indicating their widespread appeal yet possibly lesser thematic or cultural resonance specific to the U.S. audience in that period.

Conversely, the cluster of black dots represents films not yet in the top 20, many of which exhibit high relatedness density but lower ubiquity, like "Love Happens" and "21 Jump Street". This pattern may reflect a strong cultural or thematic match with the U.S. audience, suggesting potential for future popularity gains, particularly when these titles are recommended or promoted.

**Fig 7:**
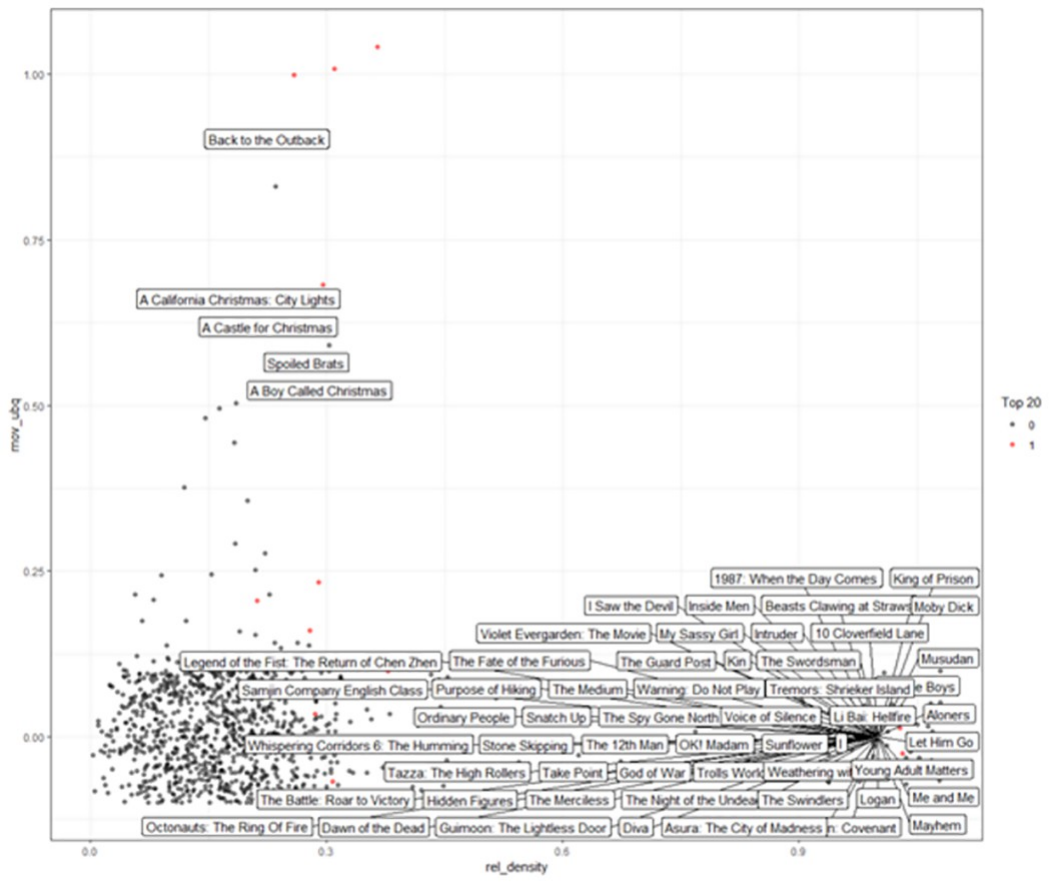
Recommendation to TOP20 high probability of entry in South Korea, December 2021.

Fig 7 offers a visual representation of the potential for various films to enter the top 20 list in South Korea for December 2021, plotted by relatedness density and movie ubiquity. The red dots indicate films that have already achieved top 20 status, such as "1987: When the Day Comes" and "King of Prison", which are positioned higher on the ubiquity axis, reflecting broad appeal, possibly due to resonant cultural themes or alignment with popular trends.

The clustering of black dots towards the low ubiquity but the high related density suggests a number of films have not yet reached the top 20 but may possess the cultural specificity or targeted appeal necessary to resonate with the South Korean audience. Titles like "Whispering Corridors 6: The Humming" and "The Battle: Roar to Victory" have moderate relatedness density scores, indicating potential for local compatibility, though their ubiquity scores are lower, implying limited global popularity.

The presence of Christmas-themed content with high ubiquity but varying levels of relatedness density, such as "A Castle for Christmas", suggests a global influence on local preferences, potentially driven by the seasonal nature of the content. In contrast, locally produced or culturally resonant films such as "Samjin Company English Class" and "Purpose of Hiking" display a strong relatedness density, indicating a closer match to South Korea’s unique cultural context despite a lower ubiquity score.

In forming a content launching strategy for the South Korean market, it may be beneficial to consider these patterns of local and global appeal, possibly by promoting movies with high relatedness density to capitalize on cultural congruence, while also leveraging globally popular movies to attract a wider audience. Through a comparative analysis of Figs 6 and 7, we can recognize distinct regional differences in movie popularity and preferences, which highlights the need for tailored strategies in content promotion and release in varying markets.

## Conclusion

Based on our findings, we conclude that a movie’s relatedness to other popular movies in a country and its ubiquity across countries are important factors in predicting its likelihood of appearing in a given country’s top 20 list. These results suggest that the success of a movie in a particular country is not based solely on the movie’s qualities but also on the preferences and trends within that country’s market. It is also evident that factors such as a movie’s IMDB score, Rotten Tomatoes score, Netflix Original status, genre, and producing country are significant predictors of the movie’s likelihood of appearing in a country’s top 20 list.

The findings of this study provide valuable insights for content providers like Netflix that are looking to tailor their content offerings to specific markets. They may choose to promote movies that are related to other popular movies in a given country or invest in producing movies in countries where there is high demand for certain genres. The findings also contribute to the broader literature on content consumption patterns across different cultural contexts. Research examining other variables that may influence a movie’s success in a particular country, such as marketing and distribution strategies, may be useful.

This study makes two significant contributions to the field of culture and media studies. Firstly, it constitutes the first attempt to measure country—movie related density, which is a novel index that captures the similarity between a movie and other popular movies in a given country. This index provides a new way of understanding the relationship between a movie’s content and its popularity in a particular country. Secondly, this study shows that country—movie relatedness density and movie ubiquity are important predictors of a movie’s likelihood of appearing in a given country’s top 20 list. This finding has practical implications for movie producers and distributors who can use these predictors to identify potential “jackpot” movies in specific countries. By considering these predictors along with other important factors such as a movie’s genre, production country, and critical scores, the industry can better allocate resources and increase the likelihood of producing successful movies in specific markets.
